# An Incidental Finding of Abdominal Aortic Aneurysm and Its Histology in a Dissected Human Cadaver

**DOI:** 10.7759/cureus.94998

**Published:** 2025-10-20

**Authors:** Rahul Sharma, Vikas Vaibhav, Gitanjali Khorwal, Bhamini Sharma

**Affiliations:** 1 Anatomy, All India Institute of Medical Sciences, Rishikesh, Rishikesh, IND; 2 Forensic Medicine and Toxicology, All India Institute of Medical Sciences, Vijaypur, Vijaypur, IND

**Keywords:** abdominal aortic anerysm, aortic aneurysm, cadaveric dissection, cardiovascular diseases, histopathology (hp)

## Abstract

Abdominal aortic aneurysm (AAA) is a vascular condition often associated with atherosclerotic degeneration and typically remains asymptomatic until rupture. It predominantly affects older males and carries a significant mortality risk if undiagnosed or untreated. This report describes a rare case of an infrarenal AAA observed during the routine dissection of a male cadaver aged 65-75 years at All India Institute of Medical Sciences (AIIMS), Rishikesh. The aneurysm exhibited fusiform dilatation with a maximum transverse diameter of 46.79 mm, accompanied by atherosclerotic changes, mural thrombus, and calcific deposits. Histological analysis revealed hallmark features of advanced atherosclerosis, including cholesterol clefts, medial elastic fibre fragmentation, and disorganisation of the tunica layers. Notably, bilateral anterior and posterior renal arteries, as well as multiple renal cysts, were observed, indicating anatomical variations with potential clinical relevance. The case underscores the importance of recognising vascular anomalies and the role of routine screening in preventing aneurysm-related complications. The findings provide significant insights for anatomical education, pathological findings and clinical risk assessment, especially in ageing populations.

## Introduction

An aneurysm, derived from the Greek word ανεύρυσμα (aneurysma), meaning "widening," is defined as a permanent and irreversible localised dilation of a blood vessel [1]. While technically, an aneurysm occurring in any segment of the aorta below the diaphragm can be classified as an abdominal aortic aneurysm (AAA), the term is most commonly used to refer specifically to an aneurysm located in the infrarenal portion of the abdominal aorta [1].

Abdominal aortic aneurysm is more common in men, with an estimated male-to-female ratio of 4:1 [2], contributing to about 1.3% of total mortality among men aged 65-85 years in developed nations [1]. These aneurysms are usually asymptomatic until they rupture, which is often catastrophic. Surgical intervention-either open repair or endovascular repair-is recommended for large or symptomatic aneurysms, as repair of small aneurysms has not shown significant benefit [3]. The condition is associated with the degradation of the elastic media in an atherosclerotic aorta. Although the exact mechanisms remain unclear, the development of abdominal aortic aneurysms involves inflammatory cell infiltration, neovascularisation, and the production and activation of various proteases and cytokines [1].

The diameter of the abdominal aorta normally varies based on age, sex, and body weight, and it gradually decreases from its entry into the abdominal cavity down to the iliac bifurcation. Adults normally have an aortic diameter of 16 to 18 mm for women and 19 to 21 mm for males, and cardiovascular disorders appear to be more likely to affect people whose diameters fall outside of this range [4]. The normal infrarenal aortic diameter rises from roughly 8 mm at age five to a median of approximately 21 mm at 70 years of age and beyond. It has been known for more than a century that the aortic diameter continues to increase with age [5]. An AAA rupture is linked to a high death rate; 59% to 83% of patients die before they are admitted to the hospital or undergo surgery [6]. This case report presents an incidental finding of an infrarenal AAA discovered during routine cadaveric dissection of an elderly male, with a focus on its anatomical and histological characteristics and its relevance to medical education.

## Case presentation

**Figure 1:**
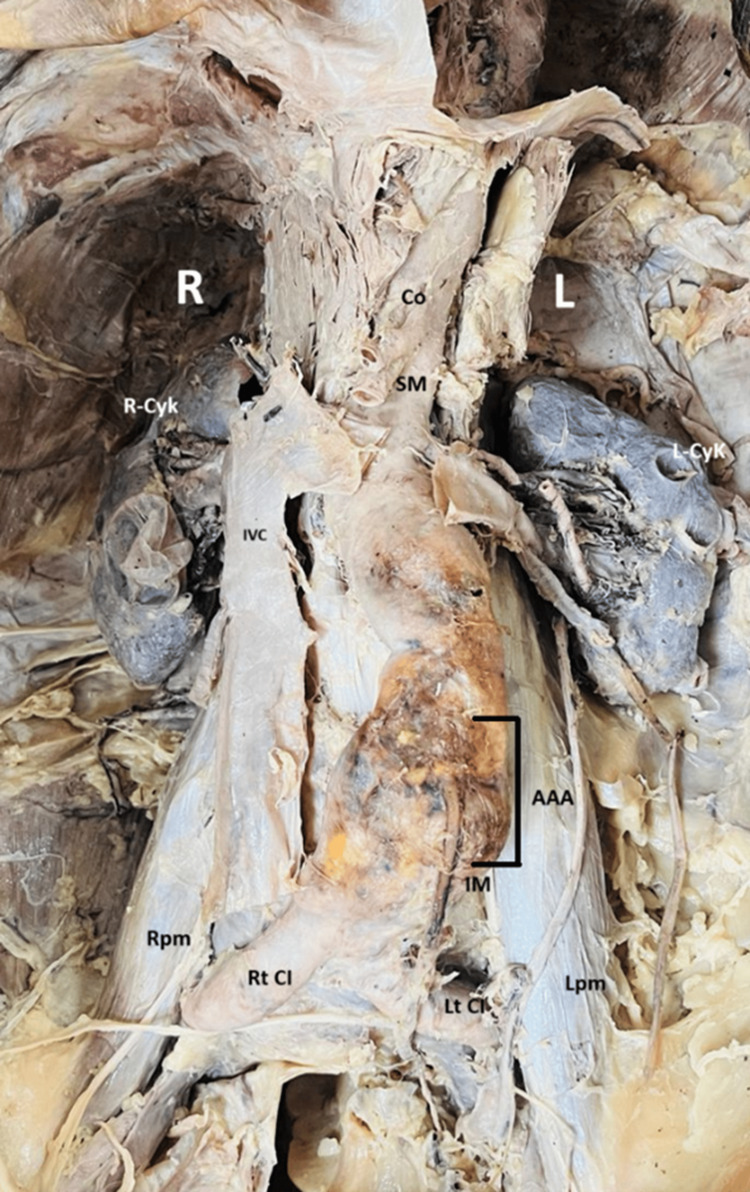
The image shows a dissection of the posterior abdominal wall showing an abdominal aortic aneurysm (AAA) and its anatomical relationships. A prominent dilation of the abdominal aorta (marked AAA) at the origin of the inferior mesenteric artery (IM), consistent with an infrarenal abdominal aortic aneurysm. Surrounding anatomical structures are labelled for orientation: Co – Celiac trunk; SM – Superior mesenteric artery; IVC – Inferior vena cava; Rt CI – Right common iliac artery; Lt CI – Left common iliac artery; R-CyK – Right kidney with Cyst; L-CyK – Left kidney with cyst; Rpm – Right psoas major muscle; Lpm – Left psoas major muscle. The aneurysmal segment appears grossly thickened and discoloured, consistent with atherosclerotic degeneration.

**Figure 2:**
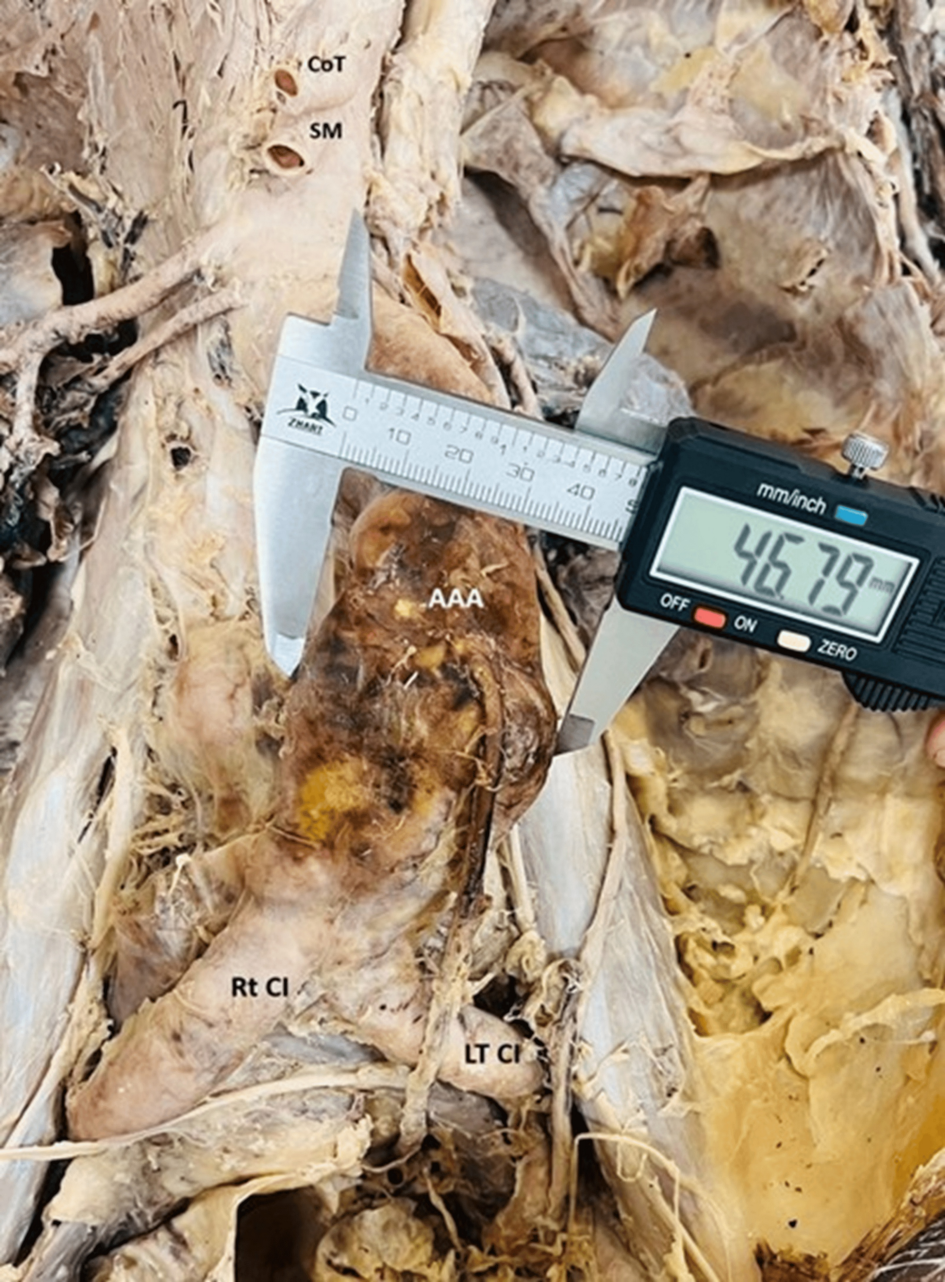
Shows measurement of an infrarenal abdominal aortic aneurysm (AAA) during cadaveric dissection. The abdominal aorta exhibits a fusiform aneurysmal dilation distal to the origin of the superior mesenteric artery (SM) and coeliac trunk (CoT). A digital Vernier caliper was used to measure the maximum transverse diameter of the aneurysm, recorded as 46.79 mm. The aneurysm is located superior to the bifurcation of the aorta into the right (Rt CI) and left common iliac arteries (Lt CI) (Note the atherosclerotic changes evident in the vessel wall showing discolouration through yellow to dark brown pigmentation of the aortic wall)

**Figure 3:**
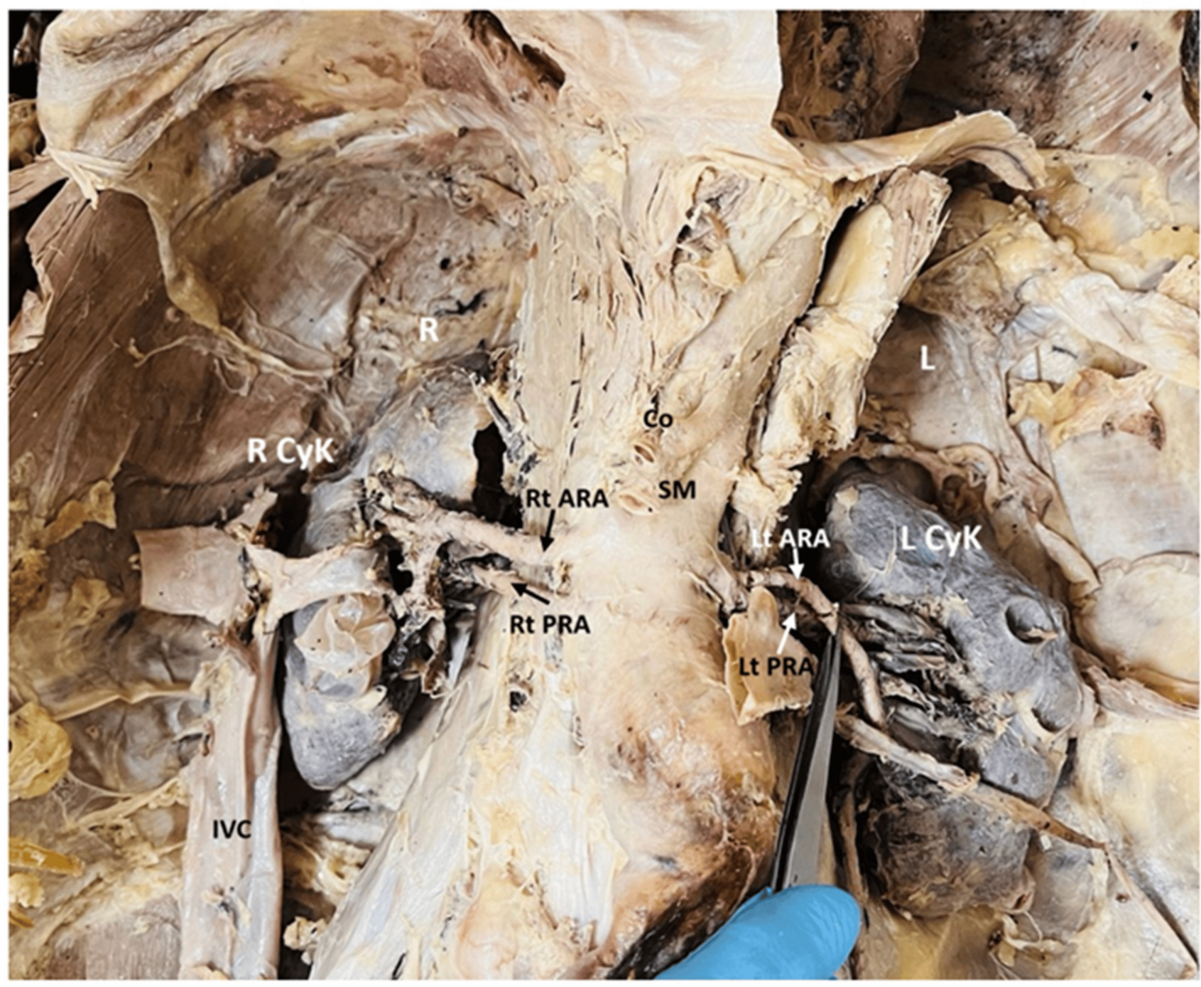
Shows the right anterior renal artery (Rt ARA) and right posterior renal artery (Rt PRA) arising directly from the abdominal aorta, with a similar pattern observed on the left side (Lt ARA, Lt PRA). These arteries are visualised branching toward their respective kidneys. Notably, both the right (R CyK) and left kidneys (L CyK) exhibit multiple surface cysts, suggesting underlying polycystic changes. Also labelled are the celiac trunk (Co), superior mesenteric artery (SM), and inferior vena cava (IVC).

During routine anatomical dissection of a preserved male cadaver aged between 65-75 years in the Department of Anatomy, AIIMS Rishikesh. No comprehensive medical history was available, and the cause of death was not documented. During dissection, an abnormal dilation of the abdominal aorta was observed, indicative of an AAA. The finding was made during the exploration of the posterior abdominal wall. The aneurysm was located in the infrarenal segment of the abdominal aorta, distal to the origin of the renal arteries and proximal to the bifurcation into the common iliac arteries. Grossly, the aorta exhibited a fusiform dilatation, which appeared markedly enlarged compared to the normal calibre of the vessel, as shown in Figures 1-2. A digital Vernier caliper was used to measure the maximum transverse diameter of the aneurysm, recorded as 46.79 mm, which was greater than the suggested normal aortic diameter in various literature sources. The aneurysmal sac exhibited thinning and degeneration of the vessel wall, accompanied by evidence of atherosclerotic plaques and calcification in the surrounding regions. The vessel wall exhibited yellowish-brown discoloration and a stiffened texture. Fibrinous material and thrombus remnants were observed along the inner lumen of the aneurysm. The aneurysm appeared to exert pressure on adjacent structures, including the inferior vena cava and renal veins, and presented no evidence of rupture in the preserved cadaver. In addition to the AAA, other anatomical variations were observed, including the origin of both anterior and posterior renal arteries arising bilaterally from the abdominal aorta. Furthermore, multiple cysts were noted on the surface of both kidneys, as demonstrated in Figure 3.

Histological findings

**Figure 4:**
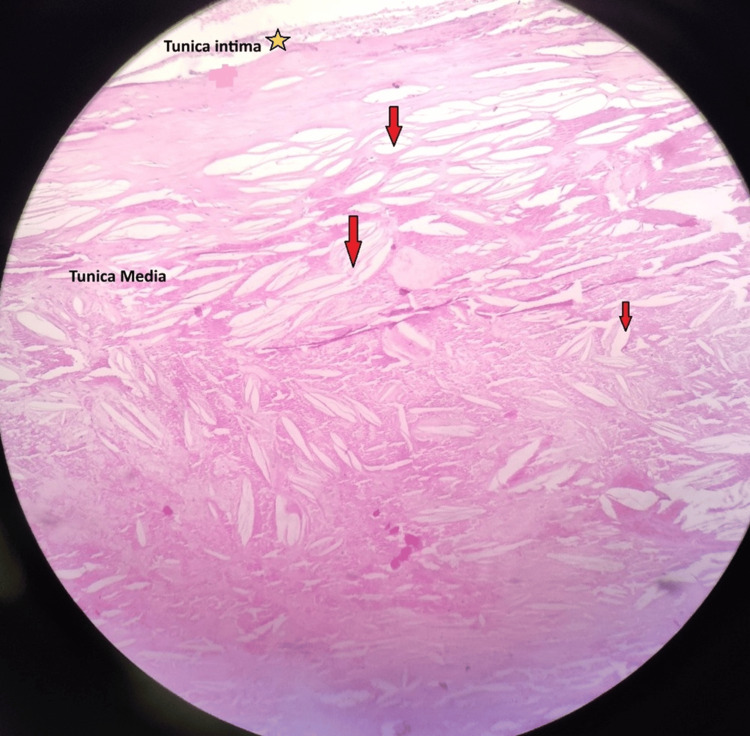
Histological section of the abdominal aorta demonstrating features of an abdominal aortic aneurysm (AAA) stained with haematoxylin and eosin (H&E under 40x). The tunica intima (★ marked) appears thinned and disrupted. The tunica media shows marked degeneration with loss of normal elastic architecture, replaced by fragments and disorganised elastic fibres. White, slit-like empty spaces scattered throughout the section are likely cholesterol clefts (marked by red arrows).

**Figure 5:**
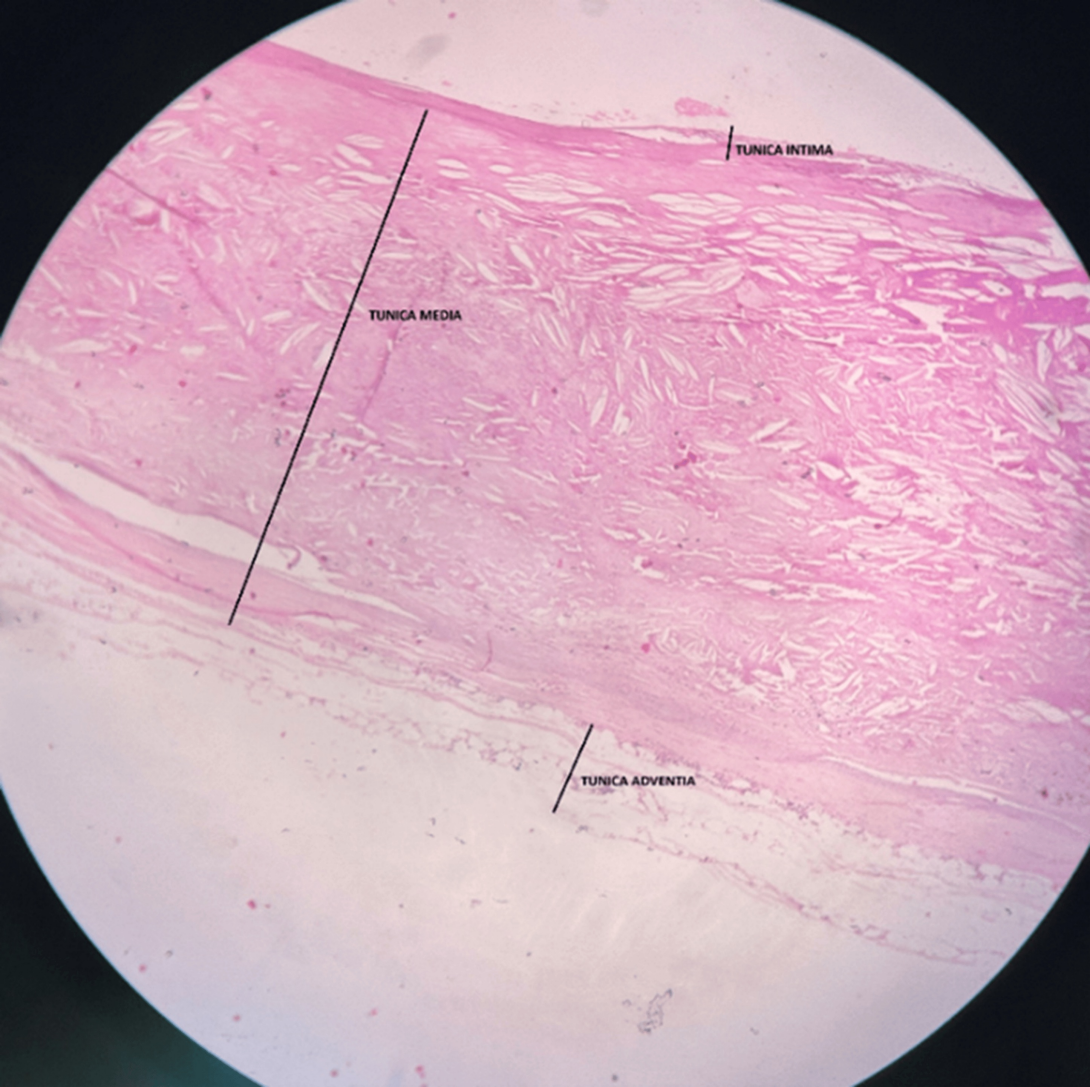
Histological section of the abdominal aorta demonstrating features of an abdominal aortic aneurysm (AAA) under (4x). The tunica media shows the slit-like empty spaces scattered throughout the section are likely cholesterol clefts, and the tunica adventitia of the image shows less organised, loosely arranged connective tissue, possibly indicating damage to the tunica layers.

Histologic findings show that the layers adjoining the lumen display relatively denser, eosinophilic (pink-stained) staining. This may represent a fibrous cap, composed of smooth muscle cells, collagen, and extracellular matrix, that forms over the atherosclerotic plaque. Lipid deposition (cholesterol clefts) was also seen. The white, slit-like empty spaces scattered throughout the section are likely cholesterol clefts. These clefts appear empty because the lipids have dissolved during tissue processing. These are the classic features of atherosclerotic plaques, as shown in Figure 4. The deeper layers show less organised, loosely arranged connective tissue, possibly indicating damage to the tunica layers. This could be due to the loss of smooth muscle cells and matrix degradation, which is commonly seen in advanced atherosclerosis, as shown in Figure 5.

## Discussion

AAAs are a critical vascular pathology, often progressing silently until a catastrophic rupture occurs. This cadaveric case of an infrarenal AAA, measuring 46.79 mm in diameter, provides valuable anatomical and pathological insights into the advanced degenerative changes associated with aneurysmal disease, mainly in older age >50 years. Histological analysis reveals hallmark features of atherosclerotic degeneration, including cholesterol clefts, medial elastic fibre fragmentation, and thinning of the intimal layer.

This case underscores the significance of early detection of cardiovascular diseases and peripheral arterial disease, as prompt diagnosis can help mitigate underlying risk factors and reduce the risk of mortality. AAAs are most often attributed to atherosclerosis, which is also the primary underlying cause of coronary artery disease and peripheral arterial disease [7]. It is most commonly seen in men between the ages of 50 and 84, with smoking being a key contributing factor to both their development and rupture [8,9]. Durojaye et al. report that the incidence is higher in men, with a prevalence ratio of approximately 4:1 compared to women [10]. Rupture of AAA remains a major cause of mortality, with rates reported to be as high as 90% when left untreated [10]. Kamboj et al. also emphasise that, in the absence of intervention, these aneurysms tend to enlarge progressively, with the risk of rupture increasing in direct correlation with their size [11,12]. It has also been observed that individuals with first-degree relatives diagnosed with an AAA have a 20% higher likelihood of developing the condition themselves [13]. Smoking is the most influential environmental factor contributing to both the development and rupture of AAAs. Individuals who have smoked less than half a pack per day for a minimum of ten years face an elevated risk (odds ratio [OR] 2.6); however, those with a history of smoking more than one pack daily for over 35 years are over 12 times more likely to develop an AAA (OR 12.1) [8]. Each additional year of smoking increases the risk of developing an AAA by approximately 4% [14]. This case highlights the critical role of cadaveric studies in medical education and anatomical exploration. It also reinforces the need for routine imaging in at-risk populations, as early detection can prevent aneurysm rupture and related morbidity.

Screening test and surveillance

Timely and effective screening can significantly reduce mortality associated with AAA rupture. The diameter of the aneurysm remains the most dependable predictor of its potential growth, rupture risk, and related mortality [7,8]. Evidence from randomized controlled trials in the U.S. and U.K. supports regular surveillance when the aneurysm is less than 5.5 cm (55 mm) in diameter. The recommended imaging schedule is as follows: for aneurysms measuring 3.0-3.9 cm, ultrasound screening every three years; for those between 4.0-4.9 cm, annual monitoring; and for aneurysms between 5.0-5.4 cm, imaging every six months. Individuals with aneurysms 5.5 cm or larger, or those presenting with symptoms such as abdominal or back pain and an aortic diameter of 3.0 cm or more, should be promptly referred to a vascular surgeon for further assessment and possible intervention [9].

Limitations

The absence of comprehensive medical history and lifestyle data limits the ability to definitively identify underlying risk factors or the progression of the disease.

## Conclusions

This case report documents an occurrence of an infrarenal abdominal aortic aneurysm identified during cadaveric dissection, offering valuable macroscopic and histological insights into aneurysm development. The case not only reinforces the established link between age-related vascular degeneration and aneurysm formation but also highlights associated anatomical anomalies, such as the presence of bilateral anterior and posterior renal arteries and multiple renal cysts. Moreover, this case underscores the importance of implementing routine vascular screening in high-risk individuals. Preventive strategies, including lifestyle modifications and routine imaging surveillance, play a crucial role in the early detection of abdominal aortic aneurysms and are essential for improving clinical outcomes - particularly in the context of an ageing global population.
